# Skin barrier-inflammatory pathway is a driver of the psoriasis-atopic dermatitis transition

**DOI:** 10.3389/fmed.2024.1335551

**Published:** 2024-03-28

**Authors:** Sitan Dong, Dongmei Li, Dongmei Shi

**Affiliations:** ^1^College of Clinical Medicine, Jining Medical University, Jining, China; ^2^Department of Microbiology and Immunology, Georgetown University Medical Center, Washington, DC, United States; ^3^Department of Dermatology/Laboratory of Medical Mycology, Jining No.1 People’s Hospital, Jining, China

**Keywords:** psoriasis, atopic dermatitis, overlap, skin barrier, tissue-resident memory T cell, antimicrobial peptides

## Abstract

As chronic inflammatory conditions driven by immune dysregulation are influenced by genetics and environment factors, psoriasis and atopic dermatitis (AD) have traditionally been considered to be distinct diseases characterized by different T cell responses. Psoriasis, associated with type 17 helper T (Th17)-mediated inflammation, presents as well-defined scaly plaques with minimal pruritus. AD, primarily linked to Th2-mediated inflammation, presents with poorly defined erythema, dry skin, and intense itching. However, psoriasis and AD may overlap or transition into one another spontaneously, independent of biological agent usage. Emerging evidence suggests that defects in skin barrier-related molecules interact with the polarization of T cells, which forms a skin barrier-inflammatory loop with them. This loop contributes to the chronicity of the primary disease or the transition between psoriasis and AD. This review aimed to elucidate the mechanisms underlying skin barrier defects in driving the overlap between psoriasis and AD. In this review, the importance of repairing the skin barrier was underscored, and the significance of tailoring biologic treatments based on individual immune status instead of solely adhering to the treatment guidelines for AD or psoriasis was emphasized.

## 1 Introduction

Psoriasis and atopic dermatitis (AD) are two common chronic immune-inflammatory diseases, each marked by distinct clinical manifestations and immunological profiles. The acute phase of psoriasis typically results from activating type 17 helper T (Th17) cells and presents with well-defined erythematous scales accompanied by mild pruritus. The acute stage of AD commences with a Th2 cell-driven inflammatory response and elevated immunoglobulin E (IgE) levels, which leads to erythema with ill-defined borders and intense pruritus. Despite these contrasting presentations, some psoriasis patients exhibit AD-like symptoms, particularly during the acute phase. However, AD can manifest psoriasiform lichenified changes in its chronic stage (1, 2). This overlap between psoriasis and AD in clinical manifestations poses diagnostic challenges and impacts treatment decisions and clinical outcomes. Moreover, the clinical manifestations of these overlapping conditions can be influenced by various factors, including the treatment regimens and immune status of individuals. Specifically, individuals with abnormalities in their skin barriers and immune responses may be more prone to exhibiting the overlapping symptoms of psoriasis and AD. The dynamic changes in local skin immunity can further contribute to the variability in clinical presentations.

Skin barriers encompass both physical and chemical components. Physical barriers are composed of keratinocytes (KCs), keratin, cornified cell envelope (CE), intercellular lipids, and skin connective structures, while chemical ones mainly comprise antimicrobial peptides (AMPs) and natural moisturizing factors (NMFs). Any impairment in these structures can predispose to a Th2 immune response.

The pathogenesis of AD and the subsequent “Atopic March” are attributed to a compromised skin barrier, which facilitates the increased penetration of external sensitized substances. The exposure of these heightened allergens triggers a systemic Th2 immune inflammation termed epithelial susceptibility (3). In addition, the interaction between Th2/Th17-related cytokines and skin structures or Kupffer cell (KC) cytokines establishes a cycle (loop) of skin barrier–inflammatory cytokine interactions. The coexistence of AD in psoriasis patients and vice versa (4–7) suggests the presence of shared pathogenic mechanisms driving the mutual conversion between the two diseases. This overlap is postulated to commence with Th2 inflammatory activation following the breakdown of the skin barrier due to genetic or other factors. In psoriasis, compromised skin barrier function may result from factors such as mechanical stimulation (intense scratching), a genetic mutation affecting epidermal barrier integrity, and the downregulation of barrier-related proteins because of dysregulated Th17-related cytokines (Supplementary Figure S1).

## 2 Keratin

Keratin, a cytoplasmic intermediate filament, serves as the primary structural protein of epidermal cells and ensures the integrity and resilience of the skin. Its proper expression orchestrated sequentially is fundamental for differentiating KCs across the various layers of the epidermis. Within the cytoplasm, keratin fibers typically aggregate into tension filaments and intricately weave a network structure. They are anchored to connective structures, such as desmosomes and half-desmosomes, and the extracellular matrix through transmembrane proteins, such as cadherins and integrins. These keratin networks interlink neighboring cells, which creates a cohesive framework. This unified structure not only shields the skin from external aggressors but also plays an important part in preserving the barrier function of the skin and safeguards against moisture loss and environmental insults.

### 2.1 Key keratins in psoriasis

As a fundamental structural protein in the epidermis, keratin10 (K10 or KRT10) plays a vital role in the hyperkeratosis and metabolic disorders of the skin. In psoriasis, various pathogenic mutations in *KRT10* have been identified in affected skin lesions, which indicates its involvement in the pathogenesis of psoriasis (8). Interestingly, the expression levels of K10 exhibit a negative correlation with the psoriasis area and severity index (PASI) (8). Moreover, therapeutic strategies that target the upregulation of K10 expression are promising in the management of psoriasis (9).

In response to skin barrier damage in healthy individuals, the expression of K1 and K10 is typically downregulated by differentiation-associated proteins, while that of K5 and K4 is upregulated by proliferative proteins. In addition, the expression of K6, K16, or K17, which is not normally expressed in KCs, is rapidly induced. Psoriasis patients often exhibit an “isomorphic reaction,” where new lesions develop in areas of damaged skin. Intriguingly, the expression pattern of keratin genes in psoriasis lesions mirrors that of damaged skin in individuals without psoriasis (10, 11), which suggests that the overexpression of K6, K16, and K17 may contribute to the excessive proliferation of KCs within psoriasis lesions (10).

The presence of the K17–T cell–cytokine inflammatory loop in psoriasis lesions is implicated in the development and exacerbation of the condition via two distinct mechanisms. First, in this loop, KCs respond to external stimuli via various pattern recognitions (PRPs), factors, and cytokine receptors. This triggers downstream signaling pathways such as extracellular signal-regulated kinase (ERK) 1/2, protein 38 (p38), transcription factors such as signal transducer and activator of transcription 1 (STAT1), STAT3, and nuclear factor-E-2 correlation factor (Nrf2), and activator protein 1 (AP-1). The binding of these transcription factors to the K17 promoter results in the upregulated expression of K17. Concurrently, cytokines produced in the epidermis further activate the Nrf2 signaling pathway, which elevates the expression of K17 (12–15). Moreover, K17 can translocate to the nucleus, which induces the expression of cytokines such as interleukin (IL)-1β and chemokines, including C-X-C motif chemokine ligand (CXCL)-1, CXCL-10, CXCL-11, and chemokine ligand (CCL)-20. These molecules accelerate the differentiation of KCs and attract more T cells and neutrophils to psoriasis lesions, which contributes to disease progression.

Second, peptides derived from K17 exhibit molecular mimicry with the M protein of β-hemolytic *Streptococcus*. These peptides act as autoantigens, polarizing naive T cells into Th1, Th17, and Th22 cell subsets, which further promotes the development of psoriasis (16). The cytokines produced by these T cells, including interferon (IFN) -γ, IL-17A, IL-22, and tumor necrosis factor (TNF)-α, activate signaling pathways in KCs, which stimulates the expression of K17s and the proliferation of KCs. Thus, the K17–T cell–cytokine inflammatory loop plays a crucial role in the pathogenesis of psoriasis, with K17 serving as a central component of this loop (12, 16, 17). Furthermore, IL-22 may contribute to this loop by inhibiting the expression of K1 and K10 through activating STAT3 (18, 19).

### 2.2 Key keratins in AD

The involvement of the keratin-inflammatory cytokine pathway is also significant in the pathogenesis of AD. To be specific, K6 acts as an alarm protein in AD and triggers the generation of pro-inflammatory cytokines and AMPs. Variations in the *KRT6* gene have been associated with the onset, severity, progression, and outcomes of psoriasis and AD (10, 20), underscoring its importance in dermatological conditions. Damage to keratin proteins disrupts skin barrier integrity and initiates a subsequent Th2-type inflammatory response. In AD, Th2-related inflammatory cytokines such as IL-4 and IL-13 downregulate the expression of key keratins such as K1 and K10, desmoglein (Dsg) 1, and desmocollin (Dsc) 1 (21). This dysregulation further exacerbates barrier dysfunction and helps perpetuate the inflammatory cascade characteristic of AD pathology.

### 2.3 Keratins for psoriasis–AD overlap

Psoriasis and AD may present distinct abnormalities in the keratin structure, but their shared consequence lies in disrupting the skin barrier. This disruption serves as a common pathway through which immune dysregulation occurs and potentially manifests as a Th2 immune disorder or a shift between Th17 and Th2 responses. Consequently, the compromised skin barrier exacerbates the chronic course and severity of psoriasis and AD individually and increases the likelihood of developing a psoriasis–AD overlap condition. In essence, the role of abnormal keratins in these dermatological conditions underlines the pivotal link between barrier integrity, immune dysregulation, and the clinical manifestations observed in patients with psoriasis, AD, and their overlap.

## 3 Cornified cell envelope

The cornified cell envelope (CE), a crucial component of the epidermis, is formed during the terminal differentiation of KCs. It consists of an insoluble tough outer membrane that forms the extensive cross-linking of various structural proteins and intercellular lipids. The CE comprises a complex network of cytoskeleton and keratin intermediate filament-related proteins. During the formation of the CE, keratin intermediate filaments and filaggrin (FLG) initially aggregate into bundles. After that, transglutaminase (TG)-1 catalyzes the connections among other structural proteins, including involucrin (IVL), loricrin (LOR), small proline-rich region proteins (SPRRs), trichohyalin, and late cornified envelope (LCE), as well as members of the S100 protein family (22–24). These interconnected proteins form a robust structural complex produced by KCs in the upper layers of the epidermis and serve as the foundation of the defense barrier of the skin.

Located at the q21.3 site on chromosome 1, the epidermal differentiation complex (EDC) encompasses a cluster of genes crucial for forming and maintaining the epidermal barrier. These genes can be categorized into three families. First, the KC envelope gene precursor family includes LOR, IVL, LCEs, and SPRRs. Second, the calcium-binding protein (S100) family contains EF-hand domains. Third, the fusion gene family is evolved from the above two, including FLG, Filaggrin-2 (FLG2), hornerin (HRNR), tripterygium hypoglaucum hutch (THH), trichohyalin-like-1 (TCHHL1), and cornulin (CRNN). The abnormal expression of any gene within the EDC, whether it encodes envelope structure proteins or enzymes involved in catalytic processes, can disrupt various differentiation stages of KCs. For instance, mutations in the FLG gene have been confirmed as a major predisposing factor for AD by triggering a Th2 immune response. Moreover, genes responsible for aggregating the keratinizing envelope within the EDC are also implicated in psoriasis (25). In psoriatic skin, a disruption in the formation of the cornified envelope (CE) could significantly compromise the barrier function of the skin (26), highlighting the importance of proper CE formation in maintaining skin health.

### 3.1 CE defects in psoriasis

In the past, it was widely believed that FLG plays a central pathogenic role in AD rather than psoriasis and psoriatic arthritis (27–30). However, the results obtained from recent studies challenge this notion, which suggests a broader role of FLG beyond AD condition and its potential association with psoriasis. For example, a study conducted in Taiwan revealed a high prevalence of the FLG P478S mutation among psoriatic patients (31). Additionally, the downregulated expression of FLG has been observed in some psoriasis patients even when identified FLG gene mutations are absent (29).

Moreover, caspase-14, a vital protease responsible for degrading FLG into NMFs, was shown to be downregulated in psoriatic hyperkeratotic skin lesions (32). The downregulation of caspase-14 indicates impaired FLG processing in psoriasis, which may contribute to the dysfunction of the skin barrier and exacerbate dry skin symptoms in psoriatic lesions. Similarly, an FLG-deficient mouse model exhibited skin inflammation dominated by Th17 responses (33). These findings collectively highlight the potential significance of FLG in psoriasis and underscore the need for further research into its role in the disease.

Mutations in six *LOR* genes have also been demonstrated in psoriasis (34), but their exact impact on the function of the CE remains unclear. Research on the LCE gene family has shown that LCE gene polymorphisms are associated with psoriasis (35, 36). The *LCE* gene family, composed of 18 members derived from *LCE1* to *LCE6*, is predominantly expressed in the skin and other keratinized epithelia. In particular, the deletion of LCE3B/C accounts for a significant proportion of psoriasis, akin to *FLG* mutations in AD (36–39). Unlike *FLG*, *LCE3B* and *LCE3C* show minimal expression in normal skin but are induced following skin damage, demonstrating their role in skin barrier repair. The inadequate post-injury repair of the skin barrier then leads to antigen penetration, which triggers toll-like receptors (TLRs) on Langerhans cell histiocytosis (LCH) or dendritic cells (DC) and subsequently activates Th17-mediated pathways involved in psoriasis (40). Further research should be conducted to examine the association of these proteins with the pathogenesis of psoriasis and AD.

CE component proteins such as FLG, LOR, and IVL are linked to Th1-, Th17-, and Th22-related cytokines. Specifically, the IL-17A-C/CAAT-enhancer-binding protein β (C/EBPB) pathway has been shown to upregulate *IVL* but downregulate *FLG* and *LOR* (41, 42). It has been found that IL-22 downregulates *FLG*, *LOR* (43), and *IVL* (42, 43) and also inhibits the expression of the EDC through the activation of the Janus kinase 1 (JAK1)-tyrosine kinase 2 (TYK2)-STAT3 pathway (43–47). Moreover, TNF-α is implicated in the downregulation of LOR expression (42). Interestingly, the level of LOR in psoriasis patients can be upregulated after using TNF-α antagonists. This suggests that TNF-α, a core pathogenic factor in psoriasis, may disrupt the skin barrier by downregulating LOR genes (48).

TG enzymes play a crucial role in maintaining the integrity of the skin barrier. TG1, TG3, and TG5 are primarily expressed in the epidermis and involved in the formation of the CE, while TG2 is predominantly expressed in the dermis and facilitates apoptosis and extracellular matrix formation. The expression levels of *TG1* and *TG2* in psoriasis patients are elevated compared to those in healthy individuals (49, 50) and positively correlated with levels of IL-6, CXCL8, and CCL20 (50). On the contrary, *TG3* is upregulated in psoriasis and acts as a protective factor by inhibiting the activation of nuclear factor kappa-B (NF-κB) through the phosphorylated STAT3-ten-eleven translocase 3 (p-STAT3-TET3) pathway, which thereby reduces skin inflammation (51, 52). Collectively, these findings underscore the intricate involvement of CE component proteins in the pathogenesis of psoriasis and highlight their potential as therapeutic targets for managing skin barrier dysfunction and inflammation in psoriatic lesions.

### 3.2 CE defects in AD

In AD, *FLG* gene mutations or expression defects (3, 53) are considered a center factor in the “out-to-in” barrier pathogenesis observed in this condition (54–56). The resulting barrier defect gives rise to subsequent local and systemic Th2 immune responses, contributes to the early onset and persistence of AD (57, 58), and manifests as symptoms such as dry skin (59), eczema, and asthma (60, 61). Th2-related cytokines can further exacerbate barrier dysfunction by downregulating the expression of *FLG* (48, 62–66). For instance, IL-4 and IL-13 activate the JAK1/JAK2-STAT6/STAT3 pathway, which inhibits the expression of the *EDC* and downregulates *FLG*, *LOR,* and *IVL* (42). Furthermore, IL-13 triggers barrier dysfunction via the downregulation of the OVOL1-FLG axis and the upregulation of the periostin-IL-24 axis (67). The absence of LOR and IVL can further promote skin antigen penetration, increase atopic susceptibility, activate Th2 response, and perpetuate inflammatory loops.

Abnormal TG expression is also observed in AD patients, although the genetic variants of TG are not considered a significant factor in AD susceptibility (68). Instead, the abnormal expression of *TG2* is associated with eosinophilic bronchitis (EB), asthma, and other atopic diseases (69). Su et al. (68) showed that *TG1* and *TG3* messenger ribonucleic acid (mRNA) are significantly increased in the skin lesions of AD patients, which indicates that they are easily upregulated after inflammatory stimulation (68). However, conflicting results regarding *TG3* expression have been reported (70), with some studies suggesting a significant reduction in both AD and non-AD lesions. In AD, TG3 and tropomyosin (TMP) can activate the Th2 response (71), and specific IgE antibodies to TG3 and TMP have been detected. Currently, no correlation has been found between *LCE* gene mutation and atopic diseases (72, 73).

### 3.3 CE molecules in the psoriasis–AD overlap

In psoriasis–AD overlap, the cytokines associated with Th1, Th17, and Th22 responses in psoriasis are beneficial to downregulating the expression of *FLG*, *LOR,* and *IVL* (41–48). This downregulation of FLG activates the Th2 immune axis (3, 53–61), further exacerbating the inflammatory response. Additionally, the Th2 immune axis *per se* can also downregulate the expression of *FLG*, *LOR,* and *IVL* (42), which thus forms a feedback loop between the epidermal barrier and inflammatory factors. This dysregulation of both the epidermal barrier and immune response aggravates the disease condition and prolongs the chronic course of AD. This reciprocal regulation between T cells and the CE may represent a critical target for understanding the inter-transformation of psoriasis and AD, as well as the chronicity of psoriasis and AD.

## 4 Epidermal connection structure-related proteins

Epidermal connection structures, including tight junctions (TJs) and anchored connections such as desmosomes and half-desmosomes, are important to maintain the structural integrity and barrier function of the skin. Key proteins involved in these connection structures, such as claudins (CLDNs) and cadherins, are fundamental to their proper function. The aberrant expression of these proteins can disrupt barrier function, which leads to persistent inflammation and skin damage. This disruption ultimately contributes to the development of conditions such as AD and psoriasis and may facilitate their interconversion.

### 4.1 TJ: CLDN

As vital components of the skin barrier, TJs are predominantly located in the lateral membranes of granular KCs. Their main function lies in sealing KCs together, which prevents the entry of external antigens and microorganisms through the skin barrier. In addition, TJs also regulate substance transport, proliferation, and differentiation, as well as the polar secretion of lipids in epidermal cells. The structure integrity of TJs relies on a family of proteins known as CLDNs, which are encoded by *CLDN* genes. Numbered from CLDN1 to CLDN27 based on their order of discovery, CLDNs form the backbone of the TJ structure.

#### 4.1.1 CLDNs in psoriasis

The expression levels of CLDN-1 and CLDN-7 are notably decreased in patients with psoriasis (74). As a member of the IL-1 cytokine family, IL-36γ is frequently over-expressed in psoriatic lesions, along with other IL-36 isomers. It has been identified that IL-36γ downregulates CLDN-1 and CLDN-7, which thereby compromises the integrity of TJs within the affected area and contributes to impaired skin barrier function (75). Additionally, the cytokines associated with the Th2/22 immune response are implicated in exerting negative effects on the expression of CLDN proteins (76).

#### 4.1.2 CLDNs in AD

CLDN-1 and CLDN-4 are key components of the TJ of the epidermis, and their absence results in embryonic lethality owing to water loss and aberrant skin phenotypes (77). Mice lacking CLDN1 displayed severe impairment in skin barrier function and reduced *CLDN1* expression, which correlates with the activation of the Th2 immune pathway, elevated serum IgE levels, increased eosinophils (EOS), and heightened susceptibility to herpes simplex virus infection (78). In the context of AD, the decreased level of CLDN-1 induces the autonomous expression of IL-1β in KCs and promotes an epidermal inflammatory response upon exposure to non-pathogenic *Staphylococci*. Reversely, the increased level of CLDN-1 has been demonstrated to enhance barrier function and alleviate inflammation (79).

#### 4.1.3 CLDNs in the psoriasis–AD overlap

Psoriasis and AD-associated cytokines have been observed to downregulate the expression of CLDNs, which disrupts the skin barrier (75, 76, 78). This dysregulation of CLDNs can lead to compromised barrier integrity and accelerate the development of Th2-type inflammation, characteristic of AD. Consequently, CLDNs emerge as a critical component involved in both Th2 and IL-1β inflammatory pathways within the spectrum of psoriasis–AD overlap. This dual involvement of CLDNs underscores their potential significance in the pathogenesis of psoriasis–AD overlap, which indicates a mechanistic link between CLDN dysregulation and the convergence of these two dermatological conditions.

### 4.2 Anchored connections: CLDNs

#### 4.2.1 CLDNs in psoriasis

CLDN proteins are vital for anchoring cellular connections, and their dysregulation is implicated in psoriasis pathogenesis. Specifically, several type I classical cadherins are associated with the development of psoriasis (80). In psoriasis vulgaris, the expression of E-cadherin, β-catenin, and T-cadherin is downregulated (81), whereas that of P-cadherin is upregulated (82). These changes may contribute to the excessive proliferation of KCs observed in psoriasis. The interaction between E-cadherin and integrin molecule αEβ7 (CD103) has been shown to aid the adhesion of lymphocytes to the skin epithelium. Abnormalities in this interaction can quicken the production of IL-17, leading to excessive epidermal hyperplasia and inflammatory leukocyte infiltration, thereby exacerbating psoriasis (83, 84). Furthermore, Dsg1, a critical component of desmosomes, is linked to psoriasis. Mice with the knocked-out *DSG1* gene exhibit the characteristics of an IL-17-skewed inflammatory signature. Current treatments that involve IL-12/23 antagonists have shown promising results in the improvement of psoriasis-related skin lesions (85).

#### 4.2.2 Cadherins in AD

Cadherin defects are indeed observed in atopic dermatitis. Skin-derived group 2 innate lymphoid cells (ILC2) express skin-homing receptors and produce type 2 cytokines upon allergen infiltration through the skin. E-cadherin can inhibit the generation of type 2 cytokines (IL-4/IL-13) after ligating to ILC2. However, the downregulation of FLG, an important protein involved in maintaining the function of the skin barrier, results in that of E-cadherin. It is one of the important characteristics of AD. Consequently, the downregulation of E-cadherin caused by that of FLG leads to the loss of inhibition of ILC2 in AD patients, which increases the production of type 2 cytokines. As a result, E-cadherin is also important in the occurrence and development of AD (86–88). In addition, Th2 cytokine (IL-4) downregulates the expression of Dsg1 and reduces the number of desmosomes, which thereby compromises the integrity of the skin barrier (88).

#### 4.2.3 Cadherins in the psoriasis–AD overlap

In the context of psoriasis–AD overlap, the downregulation of E/T-cadherin observed in psoriasis (80, 81) creates an environment conducive to producing Th2/Th17 inflammatory cytokines (83–88). These cytokines are pivotal in orchestrating the inflammatory response characteristic of both psoriasis and AD. To be specific, Th2 cytokines can downregulate the expression of Dsg1. The reduction in Dsg1 levels can lead to compromised barrier function and the skewness of subsequent inflammatory responses toward IL-17-skewed inflammation (85). Moreover, the dysregulation of cadherin expression may further perpetuate the inflammatory loop between psoriasis and AD. This interplay between multiple cadherins and inflammatory cytokines provides a potential mechanistic link for the overlap and interconversion of these two dermatological conditions.

## 5 Amps in psoriasis and AD

Chemical and physical barriers are essential components of cutaneous defense mechanisms. These barriers are primarily made up of AMPs, epidermal lipids, and NMFs (89). Among them, AMPs play a significant role in the chemical barrier of the skin. Apart from owing antimicrobial properties, AMPs are involved in various functions, including promoting cell migration, proliferation, and differentiation. They also modulate the expression of inflammatory factors and regulate the function of the skin barrier (90). In the skin, AMPs are primarily expressed constitutively or indelibly by stimuli such as microbial invasion or inflammation, KCs, and other cell types. Several key AMPs are found in human skin, including defensins, cathelicidin, ribonuclease 7 (RNase 7), psoriasin, and dermcidin (DCD). Studies have demonstrated that these AMPs involve the mechanisms underlying the development of psoriasis and AD (90).

### 5.1 Defensins

Defensins, a class of AMPs, are classified into three groups: α-defensins, β-defensins, and θ-defensins. Only α-defensins and β-defensins are expressed in humans (91). Human β-defensins (hBDs) 1–4 are expressed in leukocytes and epithelial cells (92). Despite the constitutive expression of hBD-1, hBD-2, and hBD-3, they are induced by factors such as skin barrier damage, microbial stimuli, and inflammation. Interestingly, hBD-2 is primarily resistant to Gram-negative bacteria, but hBD-3 demonstrates broad-spectrum antimicrobial activity against some microorganisms, including some multiple drug-resistant bacteria (93).

Immune disorders, barrier defects, and microbial invasion commonly found in psoriasis and AD can stimulate KCs, immune cells, and other cells to express excessive amounts of hBDs. These peptides function as antimicrobials, contribute to skin barrier repair, and modulate immune responses. Despite being elevated in the skin lesions of both psoriasis and AD patients, the expression levels of hBD-2 and hBD-3 are generally higher in psoriasis compared to AD (94). This disparity may explain why patients with AD are more prone to epidermal infections compared to those with psoriasis (95). Moreover, hBD-1 and hBD-3 are important in promoting the development and repair of TJs, crucial components of the physical barrier of the skin (96–98). In addition, hBD-3 can activate autophagy in KCs through the aryl hydrocarbon receptor (AhR) signaling pathway, which mitigates damage to the TJ barrier caused by IL-4 and IL-13 (99). It is believed that the defective expression of hBDs in AD, relative to psoriasis, is ascribed to the inhibition by Th2-type cytokines (100). Conversely, the upregulation of hBDs in psoriasis may be related to higher levels of IL-17, IL-22, and IFN-γ in the skin lesions of psoriasis patients (101). Furthermore, the modulation of T cell-mediated immune responses by hBDs enhances the generation of Th2 cytokines, IL-22, IFN-γ, and IL-10 while inhibiting the production of IL-17 (102, 103). As a result, hBDs may serve as a bridge for the interplay between Th2/Th22 and Th1/Th17 immune responses. Agents targeting AMPs may have a potential impact on the overlap and transformation of psoriasis and AD.

### 5.2 Human cationic antimicrobial protein

Human cationic antimicrobial protein (hCAP) is among the earliest AMPs discovered in mammalian skin. Derived from hCAP, LL-37 is inducibly expressed in the presence of proteases (104) and present in different kinds of tissues and cells, including epithelial cells, KCs, and macrophages (105). Similar to other AMPs, hCAP is highly expressed when cells are stimulated by trauma, infection, or inflammation and acts as an antimicrobial agent and immunomodulator (106–109).

In psoriasis, LL-37 not only directly promotes the gene expression related to psoriasis (110) but also activates TLR7/8, which further enhances this gene expression. Additionally, it serves as a central player in the intricate interplay between various aspects of skin barriers, immunity, and autophagy. Its impact on the physical barrier of the skin and innate immunity involves a few mechanisms. For example, LL-37 forms complexes with self-deoxyribonucleic acid (DNA) released from apoptotic cells, which activates plasmacytoid dendritic cells (pDCs) via TLR9 and induces the production of interferon-α (IFN-α). Then, the increased level of IFN-α triggers the activation of myeloid DCs (mDCs) and T-cells, thereby promoting the inflammatory response and the development of skin lesions in psoriasis (111).

The second crucial function of LL-37 is to uphold the integrity of epidermal permeability and antimicrobial barriers. LL-37 is stored along with other AMPs, such as hBD-2, in epidermal lamellar bodies (LBs). The disruption of the permeability barrier leads to increased lipid synthesis and elevated mRNA and protein expression of LL-37 and hBD-2 homologs in mice. Conversely, the absence of hBD-2 delays the recovery of the permeability barrier, notwithstanding increased LL-37 expression, which indicates mutual regulation between epidermal permeability and antimicrobial barriers through AMPs (112). The modulation role of LL-37 in the skin’s physical barrier results in the enhanced expression of TJ-related proteins, increased transepithelial resistance (TER), and reduced paracellular flux in the stratum corneum (SC). This process involves multiple signaling pathways and induces the expression of KC differentiation-specific proteins, which suggests that LL-37 contributes to maintaining the stability of the physical barrier while participating in cutaneous innate immunity (113). Moreover, studies have demonstrated that the restoration of the LL-37-mediated TJ barrier is associated with the activation of autophagy. In autophagy-deficient KCs and skin models, the TJ improvement induced by LL-37 was hindered, which suggests that LL-37 is capable of regulating the skin barrier by modulating autophagy (114).

In summary, the multifaceted roles of LL-37 highlight its significance in skin barrier function and immune modulation. Mast cell chemotaxis and IL-31 secretion are induced by hBDs and hCAP, which reveals their involvement in itch sensation, a common symptom in various skin diseases (115–117). Moreover, the upregulation of Th2-associated cytokines in the presence of hCAP indicates its role in promoting the inflammatory environment, potentially contributing to conditions such as psoriasis (118) and the overlap and transformation of psoriasis and AD. Considering these diverse functions, targeting LL-37 and related AMPs could provide therapeutic avenues for skin diseases featuring barrier dysfunction.

### 5.3 Psoriasin

Also known as S100A7, psoriasin plays a critical role in inflammatory cell chemotaxis, oxidative stress response, and the proliferation and differentiation of KCs. Expression levels of psoriasin are upregulated in the skin lesions of both psoriasis and AD (119–121).

Psoriasin production can be induced by an assortment of endogenous and exogenous factors and is involved in multiple signaling pathways, including AP-1, NF-κB, and STAT3. The activation of these pathways upregulates various pro-inflammatory cytokines, which directly or indirectly contribute to the pathogenesis of psoriasis and AD (122–125). In addition, several cytokines can induce the expression of S100A. IL-17, a crucial pro-inflammatory factor in psoriasis, is also important in both the acute and chronic phases of AD. IL-19, which enhances the action of IL-17A and induces IL-23, is part of the IL-23/IL-17 axis. It can also induce hBDs, which also cause the abnormal differentiation and proliferation of KCs (126). IL-1, IL-17, and IL-19 all upregulate the expression of S100A (126–128). IL-17 synergizes with IL-22 to induce the expression of S100A7, S100A8, and S100A9 (129). IL-36 also synergizes with IL-17A to induce the expression of S100A7 *in vitro* (130). In contrast to psoriasis-associated cytokines, however, Th2-associated cytokines, such as IL-4 and histamine, may hinder the expression of S100A7 in the skin (131, 132). Interestingly, Gittler et al. demonstrated an increase in S100A7, S100A8, and S100A9 genes, along with an increase in Th2/Th22 cytokines during the transition from the acute to the chronic stage of AD (129). Therefore, S100A may serve as a marker for the transition from the acute to chronic stage of AD. Additionally, chronic AD and psoriasis share overlapping immunologic and clinical features, which suggests that S100A may also play a pivotal role in psoriasis and AD.

Furthermore, akin to LL-37, S100A7 not only participates in innate immunity but also enhances the differentiation of KCs and increases the expression of epidermal differentiation markers. Similarly, it is beneficial to maintaining the stability of the skin barrier by regulating the expression of TJ-related proteins, a process modulated by glycogen synthase kinase 3 (GSK-3) and mitogen-activated protein kinase (MAPK) pathways (133). Similar to LL-37, S100A7 also serves as a crucial intersection of epidermal physical, immune, permeability, and antimicrobial barriers. The development of both psoriasis and AD involves multiple disruptions in the skin barrier and abnormalities in autophagy. Hence, AMPs such as LL-37 and S100A7 could present novel targets for treating these diseases characterized by skin barrier disorders in cases where it is challenging to distinguish between AD and psoriasis or when these conditions overlap. This approach could help avoid the direct use of potentially inappropriate immunosuppressive agents.

### 5.4 DCD

DCD, one of the AMPs with broad-spectrum activity, is produced by exocrine sweat glands and secreted onto the surface of the skin with sweat. It exerts its antimicrobial activity by inhibiting bacterial RNA and protein expression (134). Unlike hBDs and hCAP, DCD secretion is not induced by skin injury or inflammation but rather regarded as a component of the innate defense of human skin (135). Abnormal levels of DCD are implicated in the pathogenesis of psoriasis and AD. The reduced expression of FLG leads to impaired sweat transport, which results in the accumulation of DCD in sweat glands and a decrease in sweat production (134). DCD-1 L stimulates the production of Th2 cytokines (IL-4, IL-13, and IL-31) and TNF-α by KCs (136). Moreover, it significantly upregulates the activation of NF-κB (137), a pathway involved in developing psoriasis. Furthermore, DCD-derived polypeptides such as DCD (86–103) activate mast cells and induce an inflammatory reaction, which thereby contributes to the occurrence and progression of psoriasis (138).

### 5.5 RNase7

RNase 7 is one of the primary AMPs secreted by KCs and acts as an alert protein in response to the disruption of the skin barrier. Its expression exhibits a significant elevation in the lesional skin of patients with AD or psoriasis compared to healthy individuals (139). RNase 7 promotes the recognition of self-DNA by plasmacytoid dendritic cells (pDCs) and facilitates their rapid sensing of bacterial DNA. Then, activated pDCs trigger a massive release of IFN-α (139). This mechanism aligns with the IFN-α expression induced by LL-37 and hBD-2/3 and is amplified by RNase 7 (140). Notably, pDCs and IFN-α are not only of importance to combat infections but also drive the initiation and progression of psoriasis and AD (141, 142). Furthermore, IL-17A and IFN-γ induce the expression of RNase 7 in KCs synergistically via STAT3 (143). Moreover, RNase7 downregulates Th2 cytokines (IL-4, IL-5, and IL-13) through the activation of GATA binding protein 3 (GATA3) (144). The protective role of RNase7 in AD appears to be well-established, although further studies are needed to fully understand its function.

## 6 Flightless I in psoriasis and AD

Flightless I (Flii), as a member of the gelsolin superfamily of proteins, is involved in various biological processes, including embryonic development, skin barrier repair, signaling, autophagy, and cancer onset and development. Emerging evidence shows that Flii is also significant in developing AD and psoriasis.

Skin barrier damage leads to the continuous invasion of allergens, which triggers immune activation and the development of immune-inflammatory skin diseases. Flii proteins act as negative regulators in the repair of skin barrier damage. With the over-expression of Flii in mice, the formation of hemidesmosomes is impaired, which affects the adhesion and migration of KCs (145). The over-expression of Flii in embryos decreases the expression of CLDN-1 and zonula occludens-2 (ZO-2), which are proteins associated with TJs (146). Despite being identified as a negative regulator of skin barrier repair, the exact mechanism by which Flii operates remains unclear and requires further investigation.

Elevated Flii expression has been observed in the skin lesions of patients with psoriasis. It has been shown that the use of neutralizing antibodies against Flii attenuates the inflammatory response induced by imiquimod in psoriasis mice (147). Regarding the fundamental role of the TLR4-NF-κB pathway in the pathogenesis of psoriasis (148), Flii may interfere with the binding of TLR4 to myeloid differentiation primary response protein 88 (MyD88), which thereby inhibits the NF-κB pathway (149). Resultantly, this leads to a reduction in the release of downstream inflammatory factors and a decrease in psoriasis symptoms.

In an ovalbumin (OVA)-induced mouse model of AD, the over-expression of Flii results in a Th2-skewed response that exacerbates the inflammatory response. Conversely, Flii heterozygous knockout mice exhibit significant Th1 immunoreactivity and reduced severity of AD and tissue inflammation (150). It was hypothesized in this study that Flii serves as a target protein contributing to the transition and overlap of psoriasis and AD. In psoriasis patients with epidermal over-expressing Flii, the disruption of the skin barrier promotes Th2 activation, which potentially causes the transition from psoriasis to AD. Further investigation into the intrinsic mechanism of the interaction between Flii and Th cells can provide valuable insights, which may represent a potential therapeutic target for skin inflammatory diseases featuring skin barrier dysfunction.

## 7 Autophagy in psoriasis and AD

Also called type II programmed cell death, autophagy is a cellular process in which damaged or aged macromolecules and organelles are degraded by lysosomal enzymes for self-digestion when cells are under external stress (151). It is a normal physiological process in the differentiation of KCs, regulating the inflammatory response and repairing the epidermal barrier (152). Nevertheless, the dysregulation of autophagy of KCs is also involved in the pathogenesis of psoriasis, AD, and other autoimmune skin diseases (153). Defective autophagy affects the differentiation of KCs, disrupts the skin barrier, and triggers inflammation, which leads to the increased production of inflammatory factors (154). Of note, a moisturizer with strong autophagy-stimulating properties has shown promising results in improving skin barrier function and alleviating itching in AD patients by promoting skin barrier restoration and inflammation control (155).

IL-17A, a key player in AD and psoriasis pathogenesis, negatively regulates autophagy and promotes inflammatory responses. KCs stimulated by IL-17 activate the phosphatidylinositol-3-hydroxy kinase (PI3K)/protein kinase B (AKT)/mammalian target of rapamycin (mTOR) signaling pathway, which inhibits the formation of autophagic vesicles and enhances autophagic flux, thereby suppressing autophagy while promoting cholesterol degradation (156, 157). Additionally, cis-Khellactone, an inhibitor of pro-inflammatory macrophages, promotes autophagy, reduces the infiltration of dermal macrophages in psoriasis, and markedly inhibits the production of IL-17A by Th17 cells (158). Thus, the inhibition of IL-17A may represent a potential therapeutic strategy for psoriasis and psoriasis-associated dyslipidemia by alleviating autophagy inhibition. Furthermore, metformin, a medicine commonly used to treat diabetes, has been shown to convert Th17 to Treg through enhanced autophagy (159). By reducing the number of Th17 cells and increasing that of Treg cells, metformin effectively enhances autophagy and may offer a therapeutic benefit to Th17-mediated psoriasis. Furthermore, KCs stimulated by a combination of psoriasis-associated cytokines (TNF-α, IL-1A, IL-17A, IL-22, and oncostatin M) activate autophagic flux, which leads to recurrent psoriasis inflammation and increased skin barrier damage (157).

It has been shown that TNF-α, an important pro-inflammatory cytokine implicated in the pathogenesis of psoriasis, enhances the initiation of autophagosome formation but impairs subsequent processing, which leads to a negative impact on autophagy (160). In TNF-α-stimulated human immortal keratinocyte line (HaCaT) cells, the inhibitor of the wingless (Wnt)/β-catenin signaling pathway mitigates the pro-inflammatory and anti-autophagic effects of granulin precursor (PGRN) small interfering RNA (siRNA) (161). Moreover, both the number and activity of lysosomal components, including histone proteases D and L, were significantly reduced in KCs stimulated with TNF-α, which indicated impaired autophagy in AD and psoriasis (162, 163).

The protein sequestosome 1 (P62/SQSTM1), which acts as a selective autophagy receptor and a signaling hub, activates multiple inflammatory signaling pathways such as NF-κB and Nrf2 (160). The direct interaction between p62 and light chain (LC3) via the LC3-interacting region (LIR) domain facilitates the delivery of ubiquitinated protein aggregates to autophagic vesicles for selective autophagy (164). Increased p62 expression can upregulate various inflammatory signaling pathways associated with psoriasis and AD. In the TLR-NF-κB signaling pathway, the activation of TLR2/6 and TLR4 induces the autophagy pathway in human primary KCs and upregulates p62 expression (165). MyD88 and tumor necrosis factor receptor superfamily (TNFR)-associated factor 6, which are key signaling factors mediating TLR activation, play a critical role in autophagy development and p62 expression. Significant in the development of psoriasis and AD, Flii proteins hinder MyD88 binding to TLR4, which thus inhibits the TLR4-NF-κB pathway and cellular autophagy. Flii also disrupts selective autophagy by blocking the binding of p62 and LC3, thereby promoting the development of psoriasis and AD (166). The silencing of P62 results in the decreased expression of cytokines and AMPs in KCs, reduces NF-κB activity and decreases cell proliferation (165). In addition, the knockout of the AP1S3 gene associated with autophagosome formation leads to defective autophagy, increased p62 accumulation, and enhanced inflammation mediated by NF-κB and Nrf2 signaling pathways (167). Furthermore, the inactivation of the MAPK family in psoriasis decreases the autophagy of KCs, which correlates positively with the severity of psoriasis in patients and mouse models (168, 169). However, increased autophagy in KCs also results in the rapid degradation of proteins, including antigen proteins, despite exacerbating psoriasis and AD. This leads to increased recognition and presentation, which activates T helper cells (160). Moreover, the direct stimulation of the TCR enhances autophagy (170). Therefore, enhanced autophagy may promote T-cell survival and inflammatory responses, exacerbating psoriasis and AD.

Autophagy shares a common mechanism of action in psoriasis and AD, which signifies that abnormal cellular autophagy might play a significant role in the overlap, conversion, and development of psoriasis and AD. However, several questions remain unanswered, including whether autophagy promotes or attenuates skin inflammatory diseases, the pathways or mechanisms through which autophagy interacts with multiple immunoinflammatory factors, and how autophagy selectively promotes specific types of T cell differentiation. Controlling cellular autophagy could be a possible target for AD and psoriasis treatment.

## 8 Tissue-resident memory T cells and skin barrier interactions in psoriasis and AD

The recurrence of psoriasis and AD poses a significant challenge in treatment. It is currently proposed that the mechanism underlying the relapse of these conditions is closely related to the presence of tissue-resident memory T (TRM) within the skin barrier (171, 172). TRM leaves an “immune memory” in the skin even after the subsidence of inflammation (172). Upon the invasion of pathogens, initial T cells differentiate into effector and memory T cells, the latter of which is further classified into central memory T cells (TCM), effector memory T cells (TEM), and TRM (173). The residency and longevity of TRM within the skin are influenced by the interaction and regulation with KCs, fibroblasts, and other skin structural cells in the skin.

Unlike circulating T cells, TRM cannot migrate through the bloodstream and instead reside within skin tissues. This is primarily due to the binding of TRM to various ligands on the surface of KCs and its adherence to different structures within the skin barrier. TRM expresses specific markers such as CD69, CD103, and CD49a. CD103, the α chain of integrin αEβ7, binds to KC E-cadherin, which facilitates the adhesion of TRM to the epidermis and allows the residency of TRM in the skin (174). CD69 also contributes to the residency of TRM by downregulating the lymphoid tissue emigration pathway mediated by sphingosine-1-phosphate reporter 1 (S1PR1). CD49a binds to type IV collagen and mediates TRM residence within the basement membrane (175). In addition, the chemokine receptor 6 (CXCR6) C-X-C motif is expressed on human skin TRM cells, while its ligand CXCR16 is expressed on KCs, which enables the retention of TRM cells in the skin (176, 177). TRM cells are influenced by the epithelial immune microenvironment created by KCs and, in turn, activate and influence KCs. CD49^−^CD103^+^CD8^+^ TRM cells mediating KC activation and epidermal proliferation promote the production of chemokines and AMPs, which leads to inflammation and relapse in psoriasis (178).

While substantial evidence shows that DCs and TRM cells such as Th2/Tc2, Th22/Tc22, and Th17/Tc17 are present in large numbers in lesions after the subsidence of inflammation in AD, their specific mechanisms in the recurrence of AD require further investigation (179). Moreover, TRM can persist in skin tissues for months to years and is primarily regulated by the local microenvironment (IL-7, IL-15, and transforming growth factor-β) generated by KCs and fibroblasts (180, 181). To sum up, the interaction between TRM cells and the skin barrier plays a key role in the recurrence of psoriasis and AD (Supplementary Figure S2).

## 9 Acute and chronic phases of psoriasis and AD with their overlap and interconversion

Typically, AD has been viewed as a Th2-driven immune-inflammatory disorder. However, emerging evidence indicates that AD involves activating multiple T-cell axes at different stages. During the chronic phase of AD, clinical and pathological features converge with psoriasis, which is attributed to the infiltration of similar subpopulations of Th cells. According to recent findings, the heightened activation of Th2/Th22 occurs during the acute phase of AD, while the activation of Th1/Th17 progressively increases during the chronic phase (182). It is important to note that the transition from the acute to the chronic stage involves the persistent activation of Th2/Th22 and Th1/Th17 rather than a shift from Th2/Th22 to Th1/Th17 (129, 182, 183) (Supplementary Figure S3). Although the level of IL-17-producing cells is slightly higher in psoriasis patients than in those with severe AD, the difference shows no statistical significance (182). The presence of Th1/Th17 cells in chronic AD suggests a shared effector pathway with psoriasis, contributing to some of their clinical features and pathological similarities.

Interestingly, disorders affecting skin barrier-related factors have been reported to influence the polarity of Th cells. For instance, abnormalities in FLG may lead to Th2 polarity (57), whereas those in Dsg 1 may result in Th17 polarity (85). Consequently, variations in skin barrier impairments or disease during the development of psoriasis and AD can result in shifts in the dominance of Th cells and perpetuate a vicious cycle of skin barrier damage and inflammation. Clinically, this manifests as transformation, overlap, or exacerbation between psoriasis and AD. Future studies could investigate the impact of skin barrier-related factors and impairments in psoriasis and AD on aspects such as the rate and extent of transition between acute and chronic phases. Moreover, exploring whether biologics can be tailored based solely on immunologic type and the impact of Th1 and Th17 activation in the chronic phase of AD warrants investigation.

## 10 Conclusion

Skin barrier damage plays a crucial role in driving the progression of the spectrum of psoriasis/AD. Both psoriasis and AD involve skin barrier-inflammatory loops, contributing to disease exacerbation, overlap, and transformation. Various barrier factors, including keratin, CE, intercellular lipids, skin connective structure, and AMPs, participate in these inflammatory loops. It is speculated that the transition and overlap between psoriasis and AD are mediated through these skin barrier factors. Moreover, targeting skin barrier-associated factors may offer a more effective approach to modulating disease progression and transformation than solely focusing on inflammatory cytokines and signaling pathways. In the future, drugs targeting these skin barrier-associated factors could serve as upstream therapeutic targets to disrupt the barrier-inflammatory loop and attenuate disease progression and transformation. Importantly, it is proposed that psoriasis and AD inherently belong to the same disease spectrum. Their differences in clinical features are attributable to the predominance of T-cell axis activation under the influence of numerous factors. Hence, future treatments of psoriasis, AD, and overlapping psoriasis–AD conditions may directly target the immune activation state to select appropriate drugs and treatment modalities.

## Author contributions

SD: Writing – original draft. DL: Writing – review & editing. DS: Writing – review & editing.
