# Supplementary material for: Skin barrier-inflammatory pathway is a driver of the psoriasis-atopic dermatitis transition
Source: Front Med (Lausanne). 2024 Mar 28;11:1335551. doi: 10.3389/fmed.2024.1335551 (PMC11007107; doi:10.3389/fmed.2024.1335551)
Supplement: Supplementary file 1 [file Data_Sheet_1.docx]

Supplementary Material

## Supplementary Figures


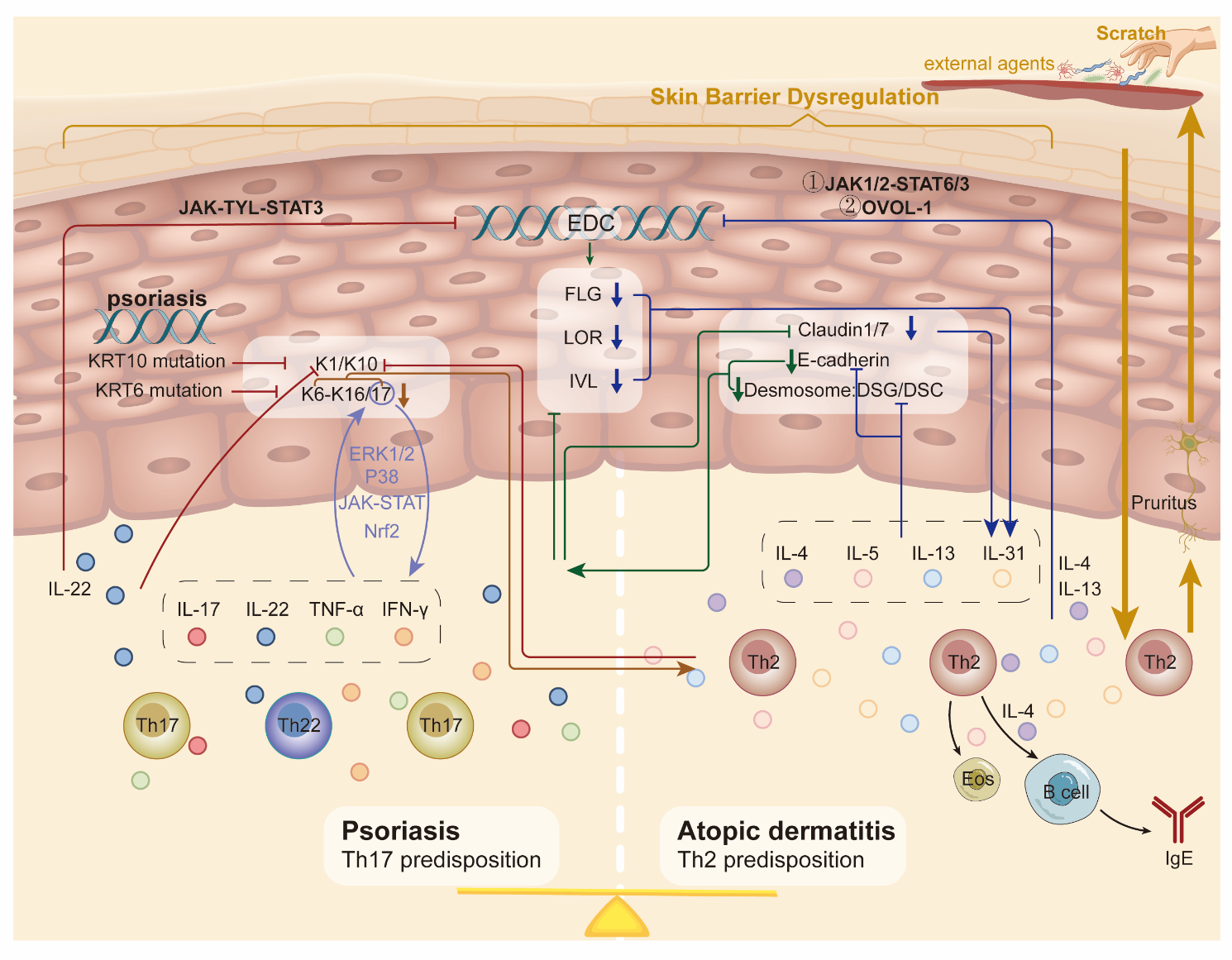


**Supplementary Figure 1.** Psoriasis and AD transform into each other through the skin barrier-inflammatory loop. Th17/Th22 (IL-17, IL-22, TNF-α, and IFN-γ, etc.) axis is activated in psoriasis, while Th2 (IL-4, IL-5, IL-13, and IL-31, etc.) axis is activated in AD. 1. Keratin (1) K1/K10: abnormal expression of K10 is caused by KRT10 mutation in psoriasis. IL-22 could down-regulate K1/K10 through STAT3 pathway. Both IL-4 and IL-13 could down-regulate K1/K10. (2) K6-k16 /K17: Both psoriasis and AD have KRT6 mutation, and abnormal expression of K6 is related to the severity and disease progression of AD. Psoriasis-related cytokines can up-regulate K17. An abnormal increase of K17 can up-regulate these psoriasis-related cytokines through signaling pathways, which form K17-T cell-cytokine inflammatory loop. The downregulation of K1/K10 and up-regulation of K17 represent hallmarks of excessive keratinocyte proliferation in psoriasis. K17 deletion shifts the cytokine profile from Th1/Th17 to Th2. These pathways are intricate and can form some Th17-TH2 transition pathways. 2. CE (FLG, LOR, and IVL): The genes that encode CE are mainly located in EDC. Th2 and Th17/Th22 related cytokines can both inhibit the transcription and translation of EDC and affect the expression of CE. Th17-related cytokines can also directly inhibit the expression of CE. The down-regulation of CE causes the activation of the Th2 axis and the formation of the Th17-Th2 transition pathway. 3. Skin connective structure: (1) Claudin: Th17-related cytokines down-regulate Claudin1/7, and the down-regulation of Claudin1/7 can promote the activation of Th2, which forms a Th17-Th2 drift pathway. (2) E-cadherin/Desmosome (DSG/DSC): Th2-related cytokines can down-regulate E-cadherin and DSG/DSC, and the down-regulation of E-cadherin and DSG/DSC can activate the Th17 axis and also form the Th2-Th17 drift pathway. 4. Psoriasis and AD-related immune factors can cause skin barrier disorders. This can in turn lead to the invasion of external allergens, activate or aggravate the Th2 immune axis, and cause the chronicity of AD or the transformation of psoriasis into AD.

Abbreviation: Atopic dermatitis: AD; T helper cells: Th; Interleukin: IL; CE: Cornified cell envelope; FLG: Filaggrin; LOR: Loricrin; INV: Involucrin; EDC: Epidermal differentiation complex; DSG: Desmoglein; DSC: Desmocollin.


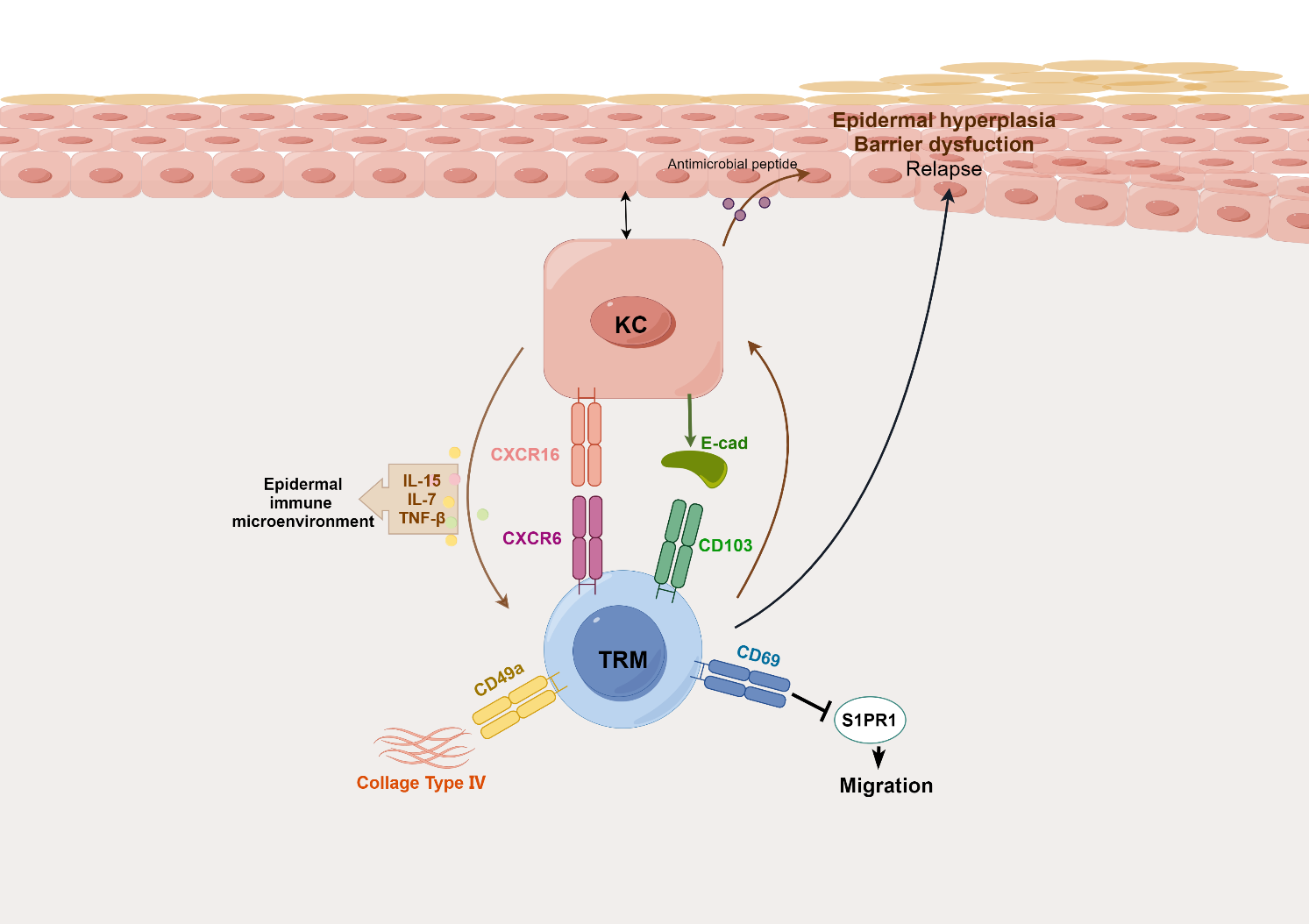


**Supplementary Figure 2. Involvement of keratinocytes (KCs) and E-cadherin in tissue resident memory T cells (TRM) -mediated PSO and AD relapse.** CD69, CD103 and CD49a and CXCR6 are commonly expressed on the surface of TRM. Binding of CD103 to KCs-produced E-calmodulin and binding of CXCR6 to KCs-expressed CXCR16 both allow TRM to reside in the epidermis. CD69 blocks the S1PR1-mediated lymphoid tissue emigration pathway, thereby blocking TRM migration. CD49a binds to collage Type IV, causing TRM to reside in the basement membrane bands of the skin as well as in mucosal sites. IL-7, IL15, and TNF-β secreted by KCs create an epidermal immune microenvironment that allows long-term survival of TRM. TRM stimulates KCs to produce antimicrobial peptides that cause epidermal hyperplasia. Different types of TRMs can produce cytokines such as IL-17/IL-23, IL-4/IL5, etc., all of which can cause epidermal barrier damage and disease relapse.


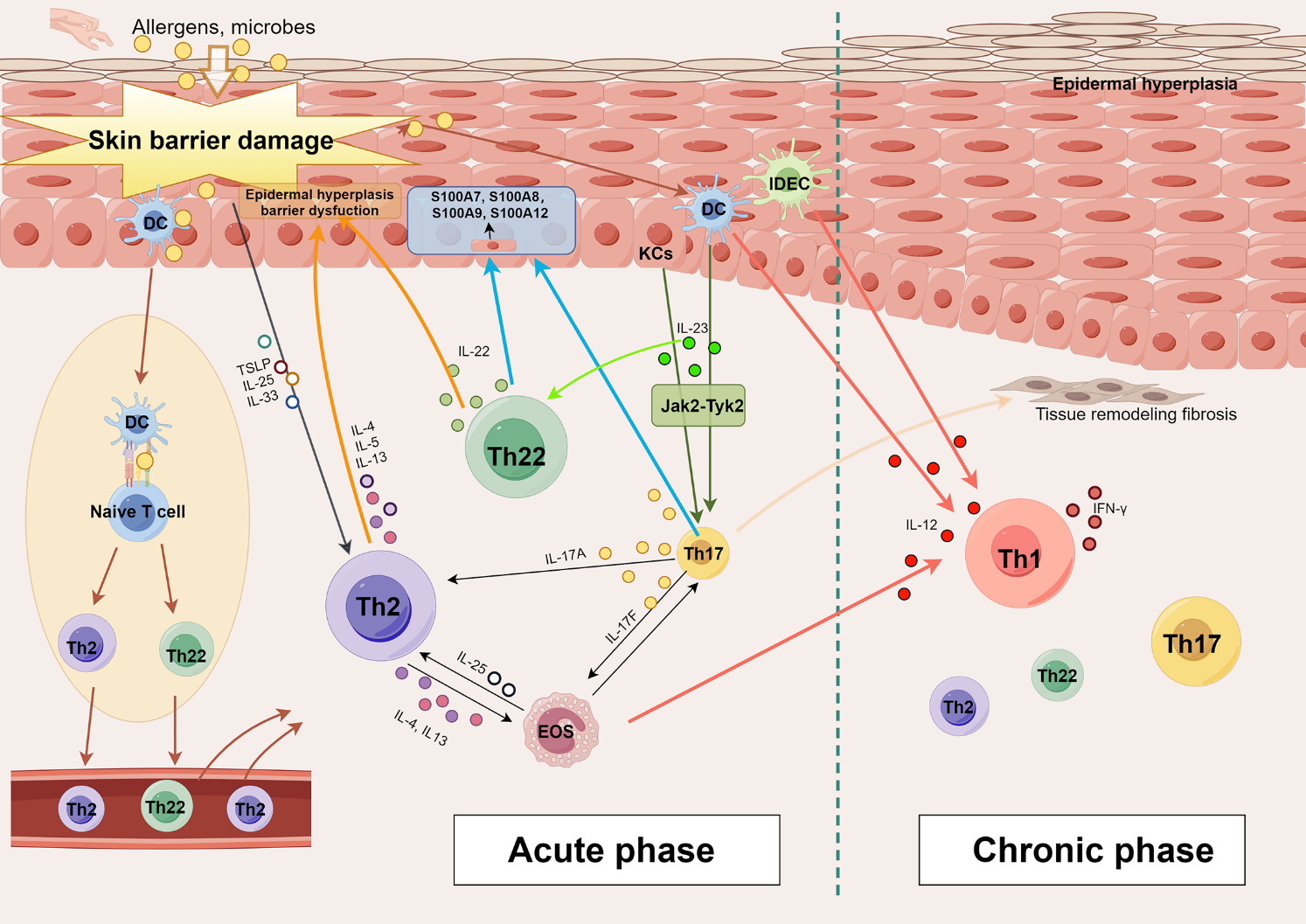


**Supplementary Figure 3. Evolution of the acute to chronic phase of atopic dermatitis (AD).** Skin barrier damage causes invasion of allergens, bacteria, etc. Antigen-presenting cells in the skin migrate to the local lymph nodes and present the antigens processed to naive T cells, causing differentiation of naive T cells to Th2 and Th22. Upon re-stimulation with antigen, cells such as Th2, Th22, and Eosinophils (EOS) migrate to the epidermis. Th2 cells releases cytokines such as IL-4 and IL5 to stimulate the secretion of IL-25 by EOS, which in turn enhances the Type 2 immune response. Moreover, keratinocytes (KCs) in barrier-disrupted epidermis produce thymic stromal lymphopoietin, IL-25 and IL-33, which also enhance the type 2 immune response. KCs in the disrupted skin barrier produce thymic stromal lymphopoietin (TSLP), IL-25, and IL-33, which also enhance type 2 immune responses. During the acute phase of AD, the Th2/Th22 axis is significantly activated, whereas the Th1/Th17 axis is not significantly activated. EOS releases IL-6, IL-1β and TGF-β to promote Th17 activation, and Th17 secretes IL-17A and IL-17F, which in turn enhance Th2 and EOS activation. Stimulated KCs and DCs secrete IL-23, which activates Th17 through the JAK2-TYK2 signaling pathway.IL-23 also enhances Th22 activation, thereby increasing the inflammatory response in AD. In addition, Th17- and Th22-related cytokines further disrupted the epidermal barrier, caused epidermal hyperplasia, and stimulated KCs to secrete antimicrobial peptides (S100A7, S100A8, S100A9, S100A100). Th17 also promotes epidermal proliferation and tissue fiber remodeling by producing IL-6, IL8, IL-11, and IL-17. IL-12 produced by EOS, DC, and inflammatory epidermal dendritic cells (IDEC) stimulates Th1 activation and drives chronicity in AD. Th1/Th17 or Th2/Th22 cell-skin barrier inflammatory loops can be formed. Therefore, the large activation of Th1/Th17 and Th2/Th22 in chronic AD lesions opens the possibility of interconversion between AD and psoriasis.
